# Genomic variant profiling in blast‐phase paediatric chronic myeloid leukaemia: Predisposing and driving alterations

**DOI:** 10.1111/bjh.20133

**Published:** 2025-05-08

**Authors:** Yvonne Lisa Behrens, Thea Reinkens, Winfried Hofmann, Amelie Gumann, Alisa Förster, Laura Gaschler, Tabita Ghete, Renate Strasser, Jennifer Espenkötter, Bernd Haermeyer, Michaela Losch, Stephanie Sembill, Zofia Wotschofsky, Stephan von Hörsten, Wolfgang Schuh, Nataliya Di Donato, Meinolf Suttorp, Manuela Krumbholz, Tim Ripperger, Brigitte Schlegelberger, Gudrun Göhring, Markus Metzler, Axel Karow

**Affiliations:** ^1^ Department of Human Genetics Hannover Medical School Hannover Germany; ^2^ Institute of Medical Genetics Carl von Ossietzky University Oldenburg Oldenburg Germany; ^3^ Department of Paediatrics and Adolescent Medicine Friedrich‐Alexander‐Universität Erlangen‐Nürnberg (FAU) Erlangen Germany; ^4^ Bavarian Cancer Research Center (BZKF) Erlangen Germany; ^5^ CCC Erlangen‐EMN: Comprehensive Cancer Center Erlangen‐EMN (CCC ER‐EMN) Erlangen Germany; ^6^ CCC WERA: Comprehensive Cancer Center Alliance WERA (CCC WERA) Erlangen Germany; ^7^ Department of Experimental Therapy, Preclinical Experimental Center University Hospital Erlangen, Friedrich‐Alexander‐Universität Erlangen‐Nürnberg (FAU) Erlangen Germany; ^8^ Division of Molecular Immunology, Department of Internal Medicine 3, Nikolaus‐Fiebiger Center University Hospital Erlangen, Friedrich‐Alexander‐University Erlangen‐Nürnberg Erlangen Germany; ^9^ Medical Faculty, Paediatric Haematology and Oncology Technical University Dresden Germany; ^10^ Amedes Genetics, MVZ Wagnerstibbe für Laboratoriumsmedizin, Hämostaseologie Und Humangenetik GmbH Hannover Germany

**Keywords:** blast phase, DNA damage repair, molecular response, paediatric chronic myeloid leukaemia, somatic variant profile, whole genome sequencing

## Abstract

Paediatric blast‐phase chronic myeloid leukaemia (CML‐BP) is a rare and serious condition. Of 231 paediatric patients enrolled in the German CML‐PAED‐II registry between January 2007 and September 2023, 25 individuals (11%) were diagnosed with CML‐BP. To identify genetic variants associated with early onset and disease transformation, we performed whole genome sequencing (WGS), deep targeted sequencing and cytogenetic analyses in 19 cases with de novo (*n* = 11) or secondary (*n* = 8) CML‐BP and sufficient available biomaterial. Copy number variants (CNVs) were more frequent than single nucleotide variants (SNVs) and more prevalent in secondary than in de novo CML‐BP. Recurrent pathogenic somatic SNVs were observed in *ABL1* (*n* = 5, 24%), *RUNX1* (*n* = 2, 12%) and *ASXL1* (*n* = 2, 12%). Nine patients (47%) carried pathogenic germline (*n* = 8) or somatic (*n* = 1) variants in either of the genes *ATM*, *CHEK2*, *FANCM*, *HERC2*, *NBN*, *RAD54B*, *RECQL4*, *SETD2* or *TP63* belonging to the DNA damage response (DDR). Within a comparison cohort of 19 patients with chronic phase paediatric CML, only one individual (5%) exhibited a pathogenic DDR germline variant. Our study provides novel pathogenetic insights into paediatric CML‐BP. The identification of pathogenic DDR‐associated germline variants suggests a genetic predisposition with potential implications for patients and families concerning cancer treatment and surveillance.

## Graphical abstract

To identify pathogenic somatic and germline gene variants associated with blast‐phase transformation and cancer predisposition in paediatric chronic myeloid leukaemia (CML), *n* = 19 patients with blast‐phase paediatric CML were analysed with whole genome and targeted panel sequencing and compared to *n* = 19 patients with chronic phase paediatric CML. In the blast‐phase cohort, recurrent pathogenic somatic variants in *ABL1*, *RUNX1*, *ASXL1* and other genes potentially driving leukaemic transformation were detected, and nearly half of the patients carried pathogenic germline (*n* = 8) or somatic (*n* = 1) variants in DNA damage response genes suggesting a cancer predisposition.

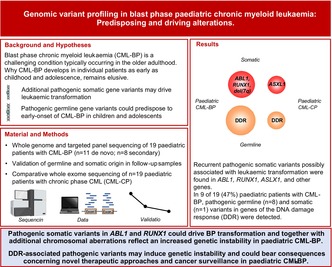

## INTRODUCTION

Chronic myeloid leukaemia (CML) is a clonal myeloproliferative disorder characterized by the somatic reciprocal balanced translocation t(9;22)(q34;q11) yielding the *BCR::ABL1* fusion gene. In the vast majority of cases, CML is diagnosed in older adulthood with a median age at onset in the sixth decade of life in Caucasians.^1^, ^2^ In childhood and adolescence, CML is rare with an annual incidence of 1 and 2.2 per million respectively.^3^ A minority of about 10% of these cases develop either a de novo or a secondary blast phase (CML‐BP)^4^, ^5^ and thus represent a particularly challenging subgroup of patients requiring intensive chemotherapy and allogeneic haematopoietic stem cell transplantation.^6^ Recent reports have demonstrated clinical and genetic differences between paediatric and adult patients with CML.^7^, ^8^, ^9^, ^10^, ^11^, ^12^ Why CML occurs in individual patients as early as childhood and adolescence remain elusive. A recent study found a generally high proportion of germline variants in paediatric compared to adult patients with CML.^13^ However, no specific predisposing genetic alterations have been described particularly in paediatric CML‐BP to date, and it is also unclear which additional genetic alterations drive transformation into the blast phase in these age groups.

Based on a whole genome level, this study aimed to comprehensively decipher likely pathogenic and pathogenic genetic variants associated with disease transformation and predisposition in paediatric patients with CML‐BP.

## PATIENTS AND METHODS

### Patient population and study design

Children and adolescents aged 0–18 years diagnosed with CML‐BP according to the current diagnostic European LeukaemiaNet (ELN) criteria^2^ and enrolled in the German national CML‐PAED‐II trial and following registry were eligible for this study. In 19 of 25 corresponding cases, sufficient deoxyribonucleic acid (DNA) from the time of CML‐BP diagnosis was available for analyses. Fourteen of these patients had been included in a previous study describing clinical features of paediatric CML‐BP.^5^ Criteria for diagnosis and treatment response were applied according to the ELN.^2^ The CML‐PAED‐II trial was conducted in accordance with the Declaration of Helsinki and approved by the institutional ethics boards of the medical faculties of the Technical University, Dresden, Germany and the Friedrich‐Alexander‐Universität Erlangen‐Nürnberg (FAU), Germany (EK282 122 006 and EK 236_18 B), and registered at EUDRACT (2007‐001339‐69) and Clinical‐Trials.gov (NCT00445822). Informed consent was obtained from patients' legal representatives and, if applicable, the patients, after providing age‐appropriate oral and written information.^14^

### Laboratory methods

All analyses were conducted in certified laboratories. Diagnoses were confirmed by central reference review. Diagnostic and response criteria were applied according to the guidelines of the ELN.^2^

The *BCR::ABL1* fusion transcript types at diagnosis and ratios during therapy were determined using standard methods^15^ and the results were depicted according to the international scale (IS) (Table S1).^16^ Fluorescence R‐banding (karyotyping) and fluorescence in situ hybridization (FISH) analyses were performed from cultured bone marrow aspirates or cultured peripheral blood as described previously.^17^, ^18^ Whenever possible, 15–25 metaphases were analysed and karyotypes were described according to the International System for Chromosome Nomenclature (ISCN 2020) (Table S1).^19^ If ≥3 aberrations are found within a clone, a karyotype is considered complex. FISH analyses on interphase nuclei were performed using a dual colour fusion probe for *BCR::ABL1* (Vysis LSI BCR::ABL Dual Colour, Dual Fusion Translocation Probe Kit; Abbott, Wiesbaden, Germany). For each patient sample, 200 interphase nuclei were analysed. We evaluated the cut‐off level for the *BCR::ABL1* probe by analysing 1000 interphase nuclei from 10 healthy donors. The cut‐off level was set to 2% for the *BCR::ABL1* FISH probe (data not shown).

Whole genome sequencing (WGS) and deep targeted sequencing with a custom panel including 148 leukaemia‐associated genes/gene regions were performed on DNA samples isolated either from the patients' blood or bone marrow aspirates according to the manufacturer's instructions (Integrated DNA Technologies, Inc., IA, USA and Illumina, San Diego, USA). WGS was carried out in all 19 cases at diagnosis of CML‐BP (Table S1). Deep targeted sequencing was complemented for 18 of the 19 patients whereas in one case, not enough further material was available. For lack of germline material from the majority of cases, the somatic or germline origin of (likely) pathogenic variants was assessed by resequencing (WGS or whole exome sequencing [WES]) of follow‐up samples in the deepest molecular response available (*n* = 17). In a comparison cohort of 19 age‐matched patients with chronic phase paediatric CML (CML‐CP) (Table 1; Table S3), WES was performed to screen for (likely) pathogenic variants in a subpanel with genes involved in DDR. The list was generated based on an NCBI search (terms: ‘DNA repair’, ‘DNA damage response’, ‘DNA replication’, ‘telomere‐associated genes’).

**TABLE 1:** Cohort characteristics.

Parameter	Variable	Total (*n* = 19 [100%])		Blast phase				Comparison cohort chronic phase (*n* = 19 [100%])	
				De novo (*n* = 11 [100%])		Secondary (*n* = 8 [100%])			
		*n*	%	*n*	%	*n*	%	*n*	%
	Female	8	42.1	5	45.5	3	37.5	6	32
	Male	11	57.9	6	54.5	5	62.5	13	68
Phenotype	Lymphoid	16	84.2	9	81.8	7	87.5	‐	‐
	Myeloid	3	15.8	2	18.2	1	12.5	19	100
*BCR::ABL1* fusion transcript	e13a2	10	52.6	5	45.5	5	62.5	3	16
	e14a2	8	42.1	6	54.5	2	25.0	11	58
	e13a2/e14a2	1	5.3	‐	‐	1	12.5	4	21
	Not known	‐	‐	‐	‐	‐	‐	1	5
Cytogenetic	High‐risk ACA	9	47.4	2	18.2	7	77.8	‐	‐
	Low‐risk ACA	3	15.8	2	18.2	1	11.1	2	11
Therapy	Dasatinib, ALL‐Ind.	9	47.4	3	27.3	6	75.0	‐	‐
	Imatinib, ALL‐Ind.	4	21.1	4	36.4	‐	‐	‐	‐
	L + TG, Ara‐c	1	5.3	1	9.1	‐	‐	‐	‐
	Imatinib only	1	5.3	1	9.1	‐	‐	‐	‐
	L + HU	1	5.3	1	9.1	‐	‐	‐	‐
	Dasatinib only	1	5.3	‐	‐	1	12.5	‐	‐
	L + AML‐Ind.	1	5.3	1	9.1	‐	‐	‐	‐
	No TKI, ALL‐Ind.	1	5.3	‐	‐	1	12.5	‐	‐
	Imatinib	‐	‐	‐	‐	‐	‐	19	100
HSCT	Yes	17	89.5	9	81.8	8	100.0	‐	
	No	2	10.5	2	18.2	‐	‐	19	100
Relapse before HSCT	Yes	4	23.5	3	30.0	1	12.5	‐	‐
	No	13	76.5	6	60.0	7	87.5	‐	‐
Relapse after HSCT	Yes	5	29.4	4	40.0	1	12.5	‐	‐
	No	12	64.7	5	50.0	7	87.5	‐	‐

*Note*: Demographic, haematological, transcript, cytogenetic, treatment and response characteristics of 19 paediatric patients presenting with de novo or secondary CML‐BP and corresponding characteristics of an age‐matched comparison cohort of (*n* = 19) paediatric patients with CML‐CP.

Abbreviations: ACA, additional cytogenetic aberration; ALL, acute lymphoblastic leukaemia; Ara‐c, cytarabin; CML‐BP, blast‐phase chronic myeloid leukaemia; Ind., induction; HSCT, haematopoietic stem cell transplantation; HU, hydroxyurea; TG, thioguanine; TKI, tyrosine kinase inhibitor.

### Bioinformatics and filter strategy

**FIGURE 1:**
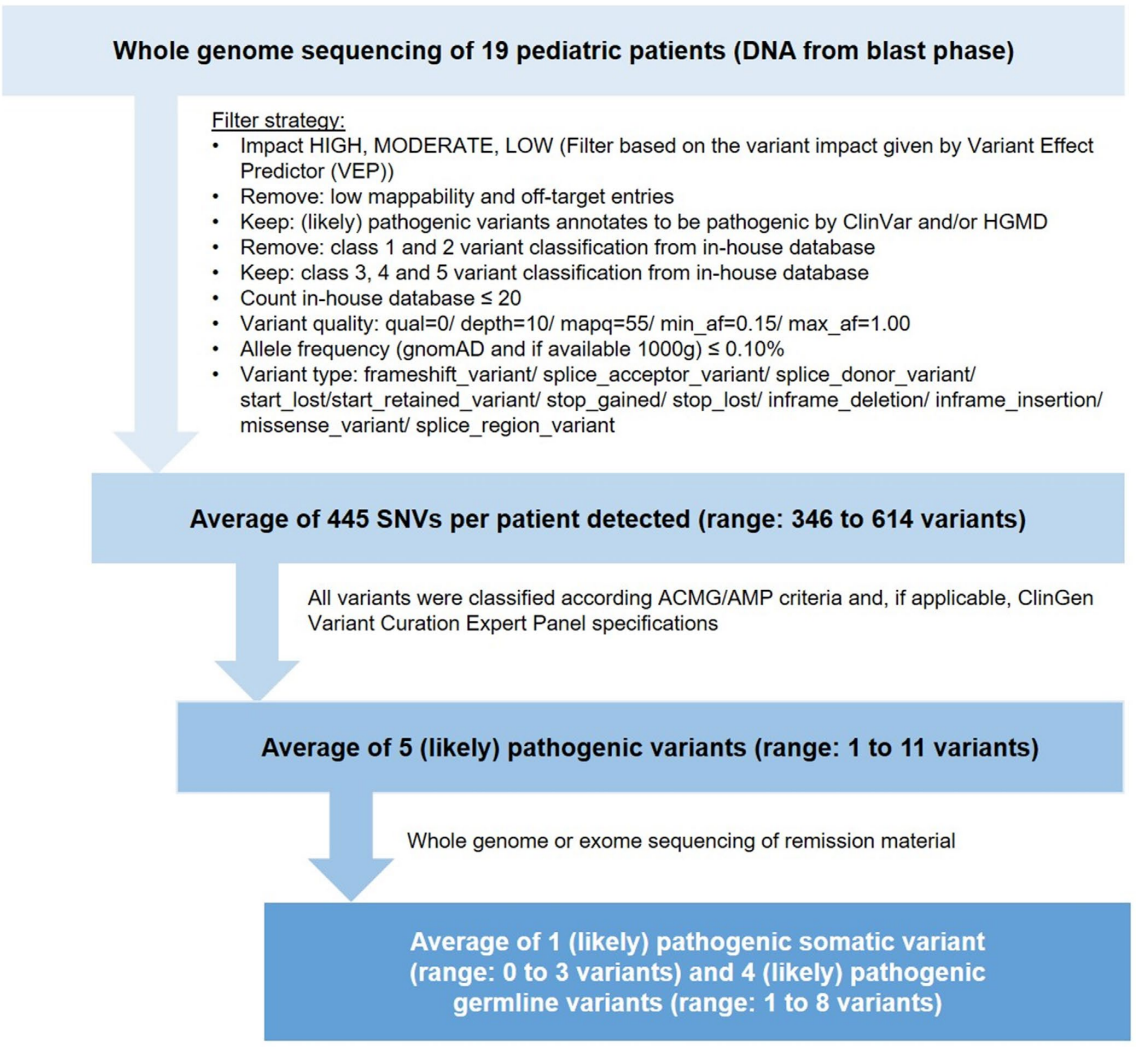
Variant filter strategy and variant evaluation of whole genome sequencing in the study cohort of (*n* = 19) paediatric patients with blast‐phase chronic myeloid leukaemia (CML‐BP).

All detected reads were aligned to the human reference genome (UCSC Genome Browser build hg38). The sequence data were processed using the megSAP analysis pipeline (https://github.com/imgag/megSAP). We used three callers for the detection of single nucleotide variants (SNVs: Illumina Dragen SNV caller, version 01.011.608.3.9.3), copy number variations (CNVs: ClinCNV, version 18.3) and structural variants (SVs: Dragen SV Caller, version 01.011.608.3.9.3). After the processing step, sequencing data were analysed using GSvar and IGV (https://github.com/imgag/ngs‐bits and http://software.broadinstitute.org/software/igv/). Different filter strategies were performed for the identification of variants. The cut‐offs/settings used for SNVs (including small InDels) are shown in Figure 1. For CNVs, the following cut‐offs/settings were used: (1) CNV size ≥50 kb, (2) filter for overlap with copy number polymorphism regions ≤0.95, (3) CNV log‐likelihood ≥12.00 and (4) CNV allele frequency ≤0.10.

Variants which passed the filtering criteria were manually confirmed using the Integrative Genomics Viewer (IGV) and classified according to the standards and guidelines of the American College of Medical Genetics and Genomics and the Association for Molecular Pathology (ACMG/AMP).^20^, ^21^ If appropriate, ClinGen Variant Curation Expert Panel specifications were applied. Furthermore, we compared the results with the variant classifications according to Horak et al.^22^ Only alterations classified as likely pathogenic and pathogenic were included in this analysis and will be subsequently described and referred to as pathogenic variants (Table S2).

### Statistical and computational analyses

Demographic characteristics are expressed as numbers and percentages or median and range for categorical and continuous variables respectively. For comparison between the subgroups, Fisher's exact test was applied. A *p*‐value <5% was considered statistically significant. The program ‘String: function protein association networks’ version 12.0 (string‐db.org) was used to analyse possible clusters of all identified pathogenic germline variants (Figure 4).

## RESULTS

### Patients and patient characteristics

In total, 231 children and adolescents with CML had been enrolled in the German national CML‐PAED‐II registry between 1 January 2007 and 30 September 2023. Twenty‐five of these patients (11%) were diagnosed with CML‐BP. In 19 cases, sufficient biomaterial was available for study analyses. Clinical and cytogenetic characteristics of these 19 paediatric patients diagnosed with de novo (*n* = 11) or secondary (*n* = 8) CML‐BP with lymphoid (*n* = 16) and myeloid (*n* = 3) phenotype included in this study are provided in Table 1.

### Variant filtering and evaluation

The application of whole genome sequencing and the analysis of the obtained data represent a major challenge in patients with haematological neoplasms. The step‐by‐step specification of detected SNVs in our study is shown in Figure 1. Initial variant filtering yielded an average of 445 SNVs per patient (range: 346–614 variants). Five variants per patient on average (range: 1–11 variants) were classified as pathogenic. Evaluation of the variant origin through resequencing of follow‐up patient samples in the deepest remission available resulted in an average of one pathogenic somatic variant (range 0–3 variants) and four pathogenic germline variants (range 1–8 variants) per patient.

### Pathogenic variant profile of CML‐BP

**FIGURE 2:**
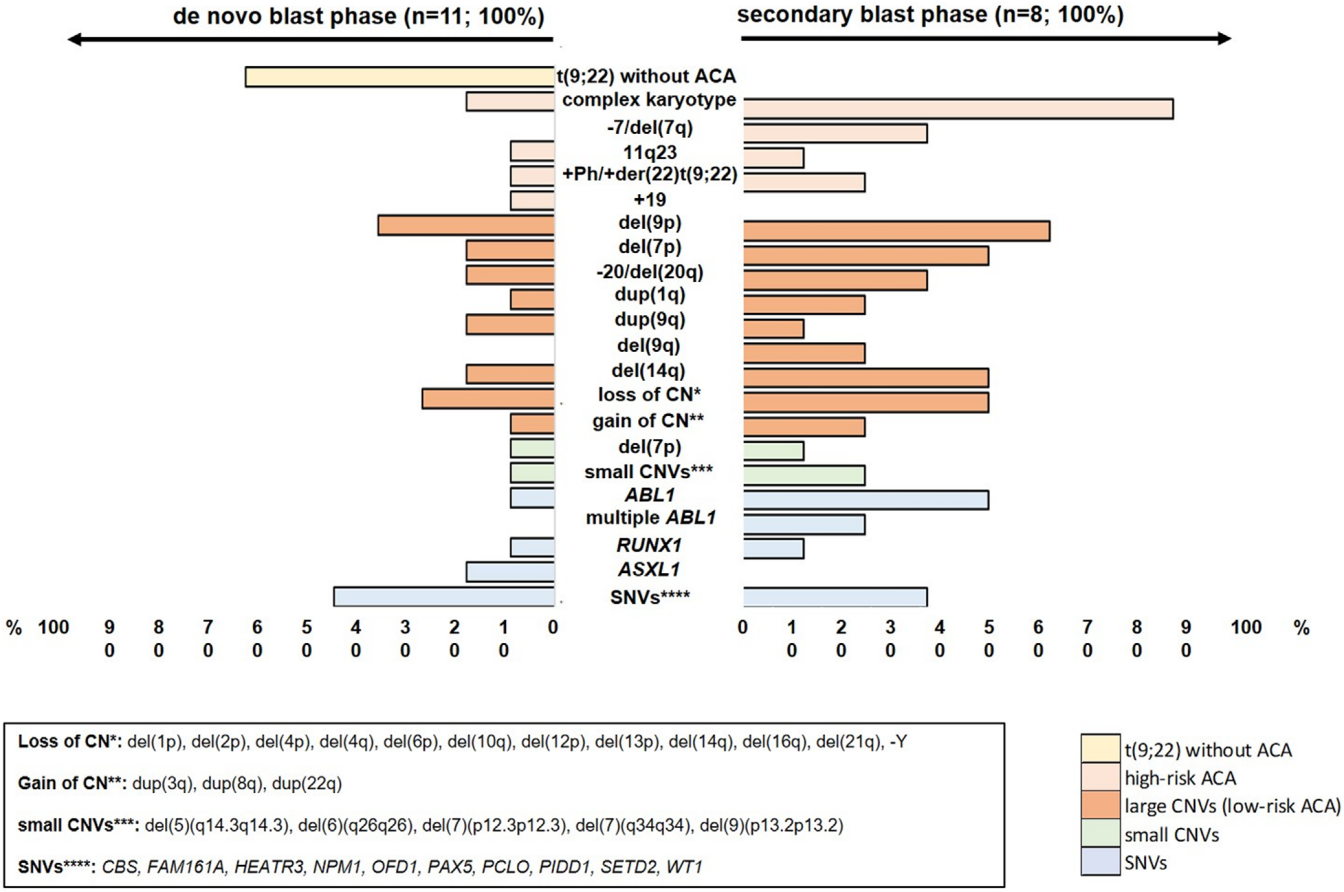
Somatic genetic landscape of de novo and secondary blast phase in CML. Frequencies of somatic variants in 19 paediatric patients with de novo (*n* = 11; left panel) or secondary (*n* = 8; right panel) blast‐phase chronic myeloid leukaemia (CML‐BP). Categories of variants [t(9;22) without ACA, high‐risk ACA, large CNVs (low‐risk ACA), small CNVs, SNVs] are highlighted in different colours. ACA, additional chromosomal aberration; CNV, copy number variant.

The landscape of somatic alterations identified through WGS, deep targeted sequencing and classical cytogenetic analysis including t(9;22)/*BCR::ABL1*, high‐risk additional chromosomal aberrations (ACAs) and large and small CNVs (including low‐risk ACAs) as well as recurrent SNVs is illustrated in Figure 2. Apart from the high‐risk ACAs (e.g. monosomy 7/deletion of chromosome 7q, or complex karyotype), recurrent copy number changes were found (e.g. deletion of chromosome 9p or 7p) (Table S1). Analysis of the whole genome sequencing data as well as of the individual karyotypes in our patient cohort regarding recombination activating gene (*RAG*)‐mediated events identified *IKZF1* deletions (*n* = 6), *CDKN2A/B* deletions (*n* = 10) and pathogenic variants in *RUNX1* (*n* = 2) in 10 patients. Overall, nine of these 10 patients showed a lymphoid phenotype. Notably, all patients with a secondary blast phase exhibited CNVs including a higher number of high‐risk ACAs compared to the patients with de novo blast phase who showed a t(9;22) without ACAs in seven cases (63%) and fewer high‐risk ACAs overall.

**FIGURE 3:**
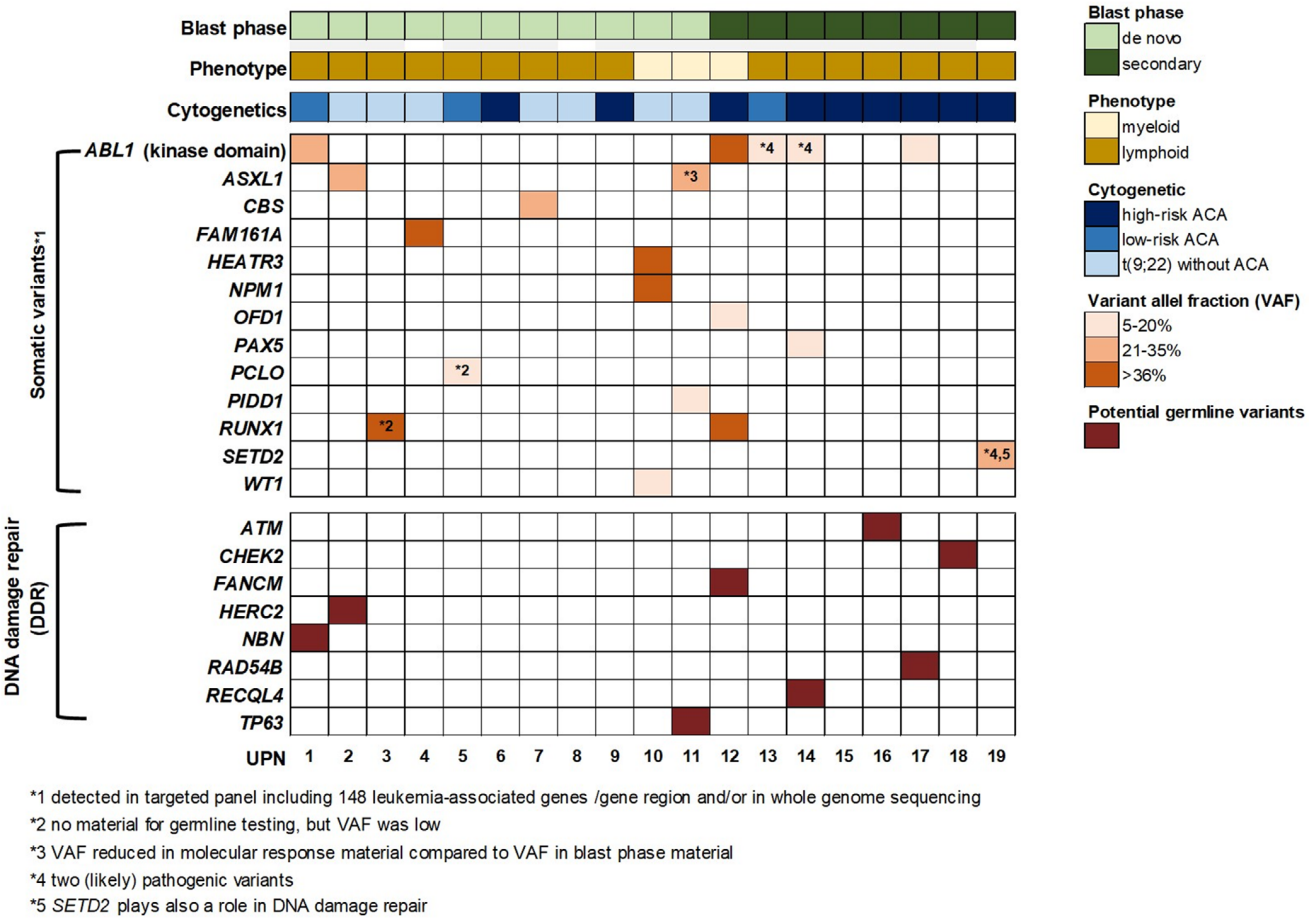
Overview of pathogenic somatic and germline variants. Categorical presentation of pathogenic somatic and germline variants detected in 19 paediatric individuals with blast‐phase chronic myeloid leukaemia (CML‐BP) (lower panel) shown in relation to blast phase, phenotype and cytogenetics with respect to additional chromosomal aberrations (ACAs) (upper panels). Graduation of variant allele frequency (VAF) is illustrated by colour intensities.

Figure 3 categorically focuses on all pathogenic somatic and germline SNVs in this cohort in relation to the de novo or secondary origin, the phenotype of the blast phase and the cytogenetic category. One or more pathogenic somatic SNVs were identified in the majority of patients (*n* = 13, 68%) in this study cohort with paediatric CML‐BP. In total, 13 different genes were involved, most of which bear a known association with different leukaemias. The three genes *ABL1* (*n* = 5, 26%), *RUNX1* (*n* = 2, 11%) and *ASXL1* (*n* = 2, 11%) were recurrently affected. Four of the five patients with pathogenic somatic *ABL1* kinase domain (KD) variants, including two cases harbouring two variants at the same time, were found in the group of patients with secondary CML‐BP. Additionally, identified somatic variants in the genes *CBS*, *FAM161A*, *HEATR3*, *OFD1*, *PCLO* and *PIDD1* lacking a known association with blood cancer are also shown in Figure 3 to ensure data availability and potential correlation with future findings.

### Pathogenic germline variants in paediatric CML‐BP

**FIGURE 4:**
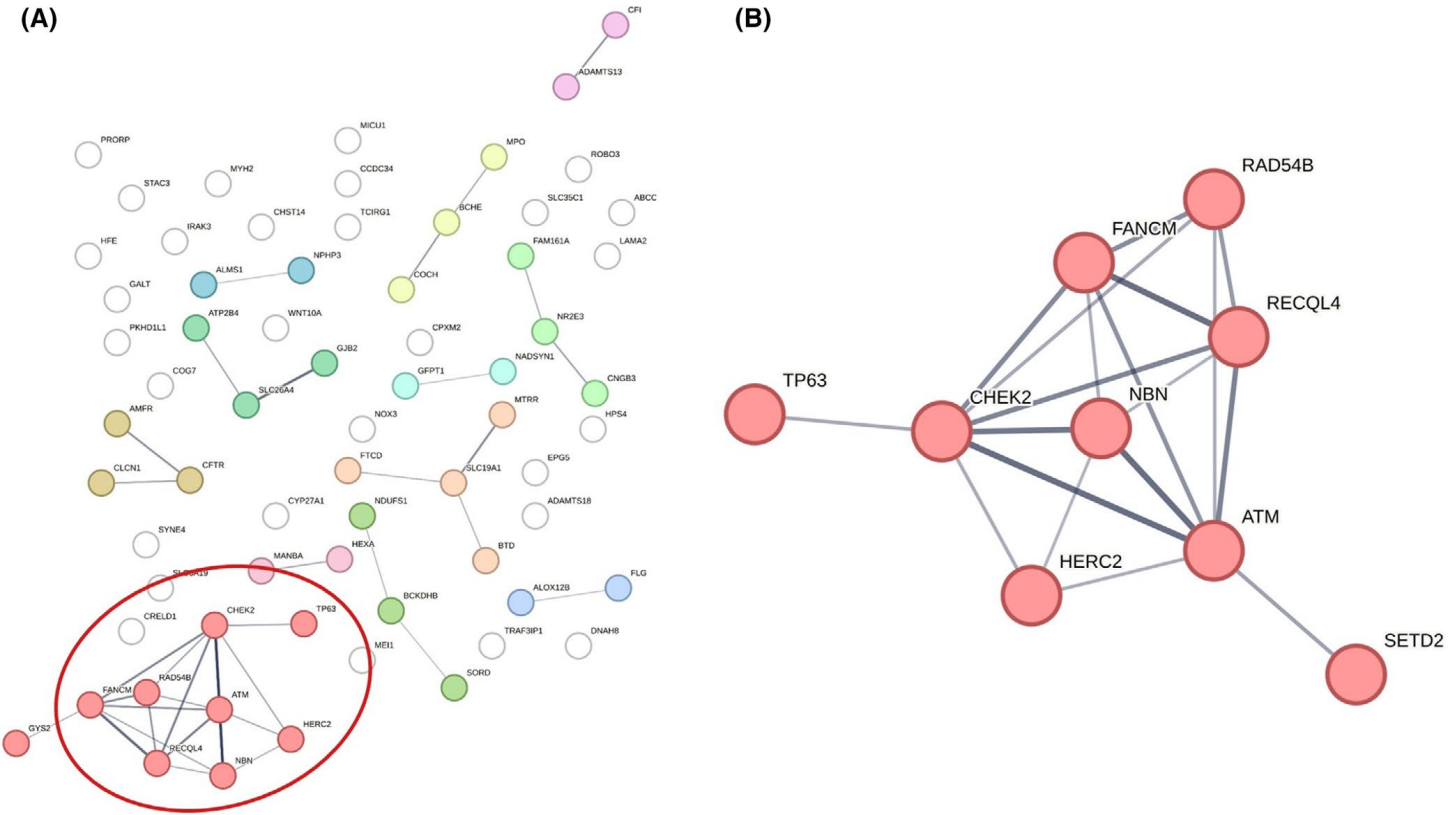
Illustration of genes and gene networks as analysed by the ‘String: function protein association networks’ version 12.0 (string‐db.org) for all pathogenic germline variants (A) and specifically for the nine pathogenic variants affecting DNA damage response genes (B). Known functional interactions between the affected genes are represented by lines, the strength of which reflects the functional association.

All variants detected by whole genome sequencing were classified and analysed following the filtering strategy depicted in Figure 1. An average of four pathogenic germline variants was detected per patient (range: 1–11 variants). Overall, 67 different genes were involved. The four genes *CFTR* (*n* = 7, 36%), *HFE* (*n* = 6, 32%), *ABCC8* (*n* = 2, 11%) and *GALT* (*n* = 2, 11%) were recurrently affected. Information on molecular function, biological process, inheritance and prevalence was collected for all genes and is available in Table S4. In addition, the STRING tool was used to analyse possible clusters of all identified pathogenic germline variants (Figure 4A). The results of this analysis show a network of genes associated with DNA damage response (Figure 4B). A deeper look in our cohort showed that nearly half of all patients (*n* = 9) carried pathogenic variants in genes encoding DDR proteins as depicted in the lower panel of Figure 3 as well as in Figure 4A,B. The affected genes comprised *ATM*, *CHEK2*, *FANCM*, *HERC2*, *NBN*, *RAD54B*, *RECQL4*, *SETD2* and *TP63* in one case each. In all but one of these patients, a germline origin of the variant could be confirmed by resequencing of remission samples. One individual harboured two distinct pathogenic variants in the *SETD2* gene which were undetectable in the follow‐up sample and therefore classified as somatic (shown in the upper part of Figure 3). In a comparative analysis of 19 age‐matched paediatric patients with CML‐CP, only a single individual was shown to carry a pathogenic germline variant in a DDR gene (*FANCC*) (Table 1; Table S3). The difference regarding the occurrence of DDR variants between the study cohort of paediatric patients with CML‐BP and the comparison group of paediatric patients with CML‐CP was significant (*p* = 0.0078, Fisher's Exact Test).

## DISCUSSION

In adult patients with CML, pathogenic somatic cytogenetic aberrations and SNVs in addition to the disease‐driving *BCR::ABL1* fusion gene have been extensively investigated in numerous studies. Various alterations were described to occur in a significant proportion of adult individuals diagnosed with CML‐CP as well as CML‐BP.^23^, ^24^, ^25^, ^26^, ^27^, ^28^, ^29^, ^30^ SNVs most commonly affected epigenetic modifier genes such as *ASXL1*, *DNMT3A* and *TET2*.^24^, ^31^ A certain proportion of these variants have also been attributed to pre‐existing age‐related clonal haematopoiesis of indeterminate potential (CHIP).^32^, ^33^, ^34^ Pathogenic somatic variants affecting the *ASXL1* gene in particular were linked to inferior molecular response characteristics and outcomes in adults with CML.^35^, ^36^ Additional alterations in genes such as *IDH1/2*, *IKZF1/3*, *RUNX1* and *TP53* have been described in adult patients with CML‐CP and CML‐BP.^30^, ^37^, ^38^, ^39^

Regarding genetic changes beyond the *BCR::ABL1* fusion gene in childhood CML, significantly less is known. Recently, we have assessed the cytogenetic landscape as well as the somatic variant profile of paediatric CML‐CP and showed that a complex karyotype and the presence of pathogenic variants in addition to the *BCR::ABL1* fusion at diagnosis were associated with inferior response characteristics.^11^, ^40^ So far, however, no data exist on the pathogenic somatic variant profile in paediatric CML‐BP, and there is no knowledge on pathogenic germline variants predisposing to the early onset of this disease in children and adolescents.

We here describe for the first time the somatic landscape of CNVs in concert with pathogenic somatic SNVs as well as underlying pathogenic germline SNVs in a paediatric cohort of children and adolescents with CML‐BP. Our analysis includes a complete dataset of whole genome sequencing, deep targeted sequencing including 148 leukaemia‐associated genes/gene regions, and cytogenetic analyses of 19 paediatric patients with CML‐BP.

The frequency of CNVs was considerably higher than the frequency of SNVs in our analysis in general and in particular in patients with secondary CML‐BP (Figure 2). This suggests a stronger influence of CNVs as disease‐driving alterations and an increased genomic instability in paediatric CML‐BP, which appears even more pronounced in patients with secondary BP as compared to patients with de novo blast phase. The detection of high‐risk ACAs comprising monosomy 7 and deletion of chromosome 7q or complex karyotype, recurrent copy number changes such as deletion of chromosome 9p or 7p, and in particular the identification of aberrant *RAG*‐mediated recombination in the genes *IKZF1*, *CDKN2A/B* and *RUNX1* in this paediatric cohort corresponded to the data from adult patients with CML.^41^, ^42^

The low number of SNVs per patient in this cohort is in line with recent comprehensive analyses demonstrating a generally lower frequency of pathogenic variants in CML than in other myeloid neoplasms with disease progression (e.g. advanced myelodysplastic syndrome).^43^, ^44^

Our analysis showed that the genes *ABL1*, *RUNX1* and *ASXL1* were recurrently affected by pathogenic somatic SNVs in this cohort of children and adolescents with CML‐BP. In concert with the absence of somatic alterations affecting *ABL1* and *RUNX1* in 90 therapy‐naive paediatric patients with CML‐CP in our previous study,^40^ these current findings propose a contribution of such pathogenic gene variants to blast‐phase transformation. This hypothesis is further supported by earlier reports on adult patients with CML showing that pathogenic variants in *ASXL1* are mostly already present in CP, but pathogenic variants in *RUNX1* and *ABL1* emerge during progression to BP.^39^, ^45^, ^46^ Moreover, Branford et al. described that *ASXL1*, *IKZF1* and *RUNX1* were frequently mutated genes in a cohort of 65 adult patients at the diagnosis of CML‐BP.^30^ As expected based on the report by Branford et al. who found that pathogenic variants in the *ABL1* KD were detected in a considerable proportion of adult patients treated with tyrosine kinase inhibitors (TKI),^30^ the pathogenic somatic SNVs detected in the *ABL1* KD in this cohort occurred mainly in paediatric patients with secondary CML‐BP underpinning a particular role of such *ABL1* KD variants for blast‐phase transformation under TKI treatment of a preceding chronic phase. Notably, a pathogenic somatic variant in *ABL1* was also found with a high variant allele frequency in one individual with de novo CML‐BP.

A further central finding of our study is the identification of pathogenic germline variants in a gene set encoding for proteins implicated in DDR, as illustrated in Figure 4. Nine of 19 individuals with CML‐BP in this cohort carried pathogenic germline (*n* = 8) or somatic (*n* = 1) variants in DDR, whereas in the age‐matched comparison cohort of 19 patients with paediatric CML‐CP, only one pathogenic DDR germline (5%) variant was found. This observation demonstrates a significant association of such variants and paediatric CML‐BP.

Germline as well as somatic DDR alterations have been associated with different haematological malignancies.^47^, ^48^ An association with CML has so far been reported only in isolated adult probands.^49^ The DDR network represents a complex system coordinating the repair of DNA damage through activation of the cell‐cycle checkpoint and other fundamental cellular pathways preventing the transmission of evolving harmful mutations and thus maintaining genome integrity.^50^ Defects in this functional group of genes have been linked to the acquisition and accumulation of DNA alterations resulting in genome instability and ultimately malignant transformation. Meanwhile, there is also knowledge of the existence of different mechanisms of synthetic lethality between DDR genes resulting in functional vulnerabilities that can be therapeutically exploited.^50^ It is well established that the dependency on alternative DNA repair mechanisms due to defective homologous recombination renders cancer cells with defects in genes associated with the BRCAness phenotype or replication stress susceptible to the targeted inhibition of poly‐(adenosine 5′‐diphosphate ribose) polymerase (PARP) or the ataxia telangiectasia and Rad 3 related‐CHK1‐WEE1 axis.^47^ In addition, the link between defective DDR, inflammation and immunity could be addressed therapeutically by the combination with immunomodulatory treatment.^47^ Such approaches would complement current concepts in the treatment of paediatric CML‐BP based on the application of TKI and chemotherapeutic elements.

The significantly lower rate of DDR‐associated pathogenic germline variants in the comparison cohort of 19 paediatric patients with CML‐CP in our study is consistent with a recent report by Krumbholz et al. The authors did not find an association of SNVs with a specific functional gene cluster such as the DDR in a cohort of paediatric patients with CML‐CP but described a rather generally elevated proportion of germline variants compared to adult patients with CML‐CP.^13^ Different from our approach, this previous study was not exclusively based on variants classified as (likely) pathogenic (i.e. class 4, 5) resulting in a comparably higher number of variants.

## CONCLUSION

Based on a WGS and deep targeted sequencing approach, this study identified recurrent pathogenic somatic variants in the genes *ABL1*, *RUNX1* and *ASXL1* driving blast‐phase transformation as well as a high number of ACAs (e.g. high‐risk ACA, small and large CNVs) underlining an increased genomic instability in paediatric CML‐BP. Our results highlight the potential predictive and therapeutic value of variant profiling in the diagnostic workup of paediatric CML‐BP. For the first time, pathogenic variants were described in genes associated with DDR in paediatric patients with CML‐BP. These latter genetic variants could support genetic instability and acquisition of aberrations and thus play a role in both blast‐phase transformation and predisposition to cancer. The identification of an underlying hereditary tumour predisposition syndrome would be of major relevance for the patients as well as for their families in the context of therapeutic options and cancer surveillance.

International collaborative networks will increase the number of enrolled patients and corroborate the findings of the present study.

## AUTHOR CONTRIBUTIONS

YLB designed the research study, performed the research, analysed the data and wrote the paper; ThRe performed the research and analysed the data; WH, AG, AF, SvH and WS analysed the data; LG, TG, RS, JE, BH, ML, SS and ZW performed the research; NDD, MS, MK, TiRi, BS, GG and MM analysed the data and wrote the paper; AK designed the research study, analysed the data and wrote the paper.

## FUNDING INFORMATION

This project was funded by the Deutsche Kinderkrebsstiftung (DKS 2021.21). YLB, TiRi and MM were supported by the BMBF MyPred consortium (01GM1911A).

## CONFLICT OF INTEREST STATEMENT

The authors declare no relevant conflict of interest.

## ETHICS APPROVAL STATEMENT

The CML‐PAED‐II trial was conducted in accordance with the Declaration of Helsinki and approved by the institutional ethics boards of the medical faculties of the Technical University, Dresden, Germany and the Friedrich‐Alexander‐Universität Erlangen‐Nürnberg (FAU), Germany (EK282 122 006 and EK 236_18 B).

## PATIENT CONSENT STATEMENT

Informed consent was obtained from patients' legal representatives and, if applicable, the patients, after providing age‐appropriate oral and written information.

## CLINICAL TRIAL REGISTRATION

The CML‐PAED‐II trial was registered at EUDRACT (2007‐001339‐69) and Clinical‐Trials.gov (NCT00445822).

## Supporting information


Tables S1–S4.

## Data Availability

The data presented in this study are available on request from the corresponding author. The data are not publicly available due to privacy and ethical restrictions.
