# Supplementary material for: Genomic variant profiling in blast‐phase paediatric chronic myeloid leukaemia: Predisposing and driving alterations
Source: Br J Haematol. 2025 May 8;207(1):141–50. doi: 10.1111/bjh.20133 (PMC12234276; doi:10.1111/bjh.20133)
Supplement: Supplementary file 1 — Tables S1–S4. [file BJH-207-141-s001.zip › bjh20133-sup-0001-Tables.docx]

**Table S1: Overview of molecular and cytogenetic results at diagnosis of blast phase (n=19).**

|  |  |  | **Molecular and cytogenetic analysis at blast phase** | | | | | |
| --- | --- | --- | --- | --- | --- | --- | --- | --- |
| **UPN** | **Age at BP** | **De novo or secondary blast phase (BP) / phenotype** | ***BCR::ABL1* fusion transcript (*BCR::ABL1* transcript ratio at diagnosis of BP (%))** |  | **Karyotype at blast phase** | **Targeted IDT panel at blast phase** | **SNVs** | **CNVs** |
| 1 | 10 | de novo/ lymphoid | e14a2 (>100) |  | 46,​XY,​t(9;22)(q34;q11)[6]/45,​idem,​-Y[14].​nuc ish 9q34(ABLx3),​​22q11(BCRx2)(BCR con ABLx1)[88/100] | *ABL1*:c.1375G>A p.(Glu459Lys) | *ABL1*:c.1375G>A p.(Glu459Lys)  *ALOX12B*:c.340C>T p.(Arg114Trp) *MANBA*:c.731C>G p.(Ser244*) *NBN*:c.657_661del p.(Lys219Asnfs*16)  *PRORP*:c.1064_1065del p.(Ile355Argfs*11) | del(9)(p13.1p24.3)/ *CDKN2A/B* deletion#  dup(9)(q21.12q34.3)  -Y |
| 2 | 4 | de novo/ lymphoid | e14a2 (73) |  | 46,XX,t(9;22)(q34;q11)[15] | *ASXL1*:c.1773C>A p.(Tyr591*) | *ASXL1*:c.1773C>A p.(Tyr591*)  *CFTR*:c.1210-34TG [12]T[5] p.? *COCH*:c.263G>T p.(Gly88Val) *GJB2*:c.35del p.(Gly12Valfs*2) *HERC2*:c.1285G>A p.(Gly429Arg)  *HFE*:c.187C>G p.(His63Asp) *MEI1*:c.938del p.(Pro313Glnfs*36) | / |
| 3 | 4 | de novo/ lymphoid | e13a2 (>100) |  | 46,XY,t(9;22)(q34;q11)[15] | no material | *CCDC34:*c.498+1G>A p.? *CFTR:*c.1210-34TG [12]T[5] p.? *CYP27A1:*c.1342C>T p.(Arg448Cys) *DNAH8:*c.12496C>T p.(Arg4166*) *LAMA2:*c.6196A>T p.(Lys2066*)  *RUNX1*:c.319C>T p.(Arg107Cys)# | del(7)(p12.1p12.1)/ *IKZF1* deletion# |
| 4 | 14 | de novo/ lymphoid | e14a2 (>100) |  | 46,XX,t(9;22)(q34;q11)[20] | / | *CFTR:*c.1210-34TG[12]T[5] p.?  *FAM161A*: c.1751+1G>A p.?  *SYNE4*:c.129-1G>T p.? | / |
| 5 | 15 | de novo/ lymphoid | e13a2 (>100) |  | 46,​XY,​t(9;22)(q34;q11)[3]/46,​XY,​ider(9)(q10)t(9;22),​der(22)t(9;22)[12].nuc ish 9q34(ABLx2~3),​22q11(BCRx2)(BCR con ABLx1)[92/100] | / | *CHST14:*c.653G>T p.(Arg218Leu) *FLG:*c.1501C>T p.(Arg501*)  *PCLO*:c.7325dup p.(Leu2443Valfs*15)  *STAC3*:c.862A>T p.(Lys288*) | del(9)(p11.1)/ *CDKN2A/B* deletion# dup(9)(q12q34) dup(22)(q11.22) |
| 6 | 4 | de novo/ lymphoid | e14a2 (37) |  | 46,​XX,​t(9;22)(q34;q11)[12]/45,​XX,​-7, der(9)t(7;9)(q11;p11),​t(9;22)(q34;q11),​del(11)(q14q22),​del(20)(q11q13)[3].​nuc ish 9q34(ABLx3),​22q11(BCRx2)(BCR con ABLx1)[90/100] | / | *NDUFS1*:c.968T>G p.(Leu323Arg) *SLC19A1*:c.274T>C p.(Tyr92His) | del(7)(p11.2)/*IKZF1* deletion# del(9)(p13.1)/ *CDKN2A/B* deletion# del(11)(q21q24.1) +19 |
| 7 | 4 | de novo/ lymphoid | e13a2 (59) |  | 46,​XY,​t(9;22)(q34;q11)[14]/46,​XY[1].nuc ish 9q34(ABL1x3),​22q11(BCRx2)(BCR con ABL1x1)[92/100] | / | *CBS*:c.833T>C p.(Ile278Thr)  *GFPT1*:c.686-2A>G p.? *GYS2*:c.547C>T p.(Gln183*) *HFE*:c.187C>G p.(His63Asp) *NOX3*:c.1159G>A p.(Gly387Arg) *NR2E3*:c.227G>A p.(Arg76Gln) | / |
| 8 | 14 | de novo/ lymphoid | e13a2 (>100) |  | 46,​XX,​t(9;22)(q34;q11)[15].nuc ish 9q34(ABLx2~3),22q11(BCRx2~3)(BCR con ABLx1~2)[97/100] | / | *BCKDHB*:c.1149T>A p.(Tyr383*) *CFTR*:c.1210-34TG [12]T[5] p.? *CLCN1*:c.950G>A p.(Arg317Gln) *CRELD1*:c.927T>A p.(Cys309*) *NPHP3*:c.3373C>T p.(Arg1125*) | del(7)(p12.2) del(9)(p21.1p22.1)/ *CDKN2A/B* deletion# dup(9)(q34.11) del(14)(q11.2q12) del(20) (q13.13q13.2) dup(22)(q11.23) |
| 9 | 16 | de novo/ lymphoid | e14a2 (>100) |  | 46,​XY,​?add(Y)(q12),​t(9;22)(q34;q11),​der(16)t(1;16)(q21;q23)[7]/46,​idem,​add(2)(p24)[7]/ 45,​idem,​der(4)t(4;13)(q21;q12),​-13[2]/46,​XY[4].nuc ish 9q34(ABLx3),​22q11(BCRx2)(BCR con ABLx1)[52/100] | / | *ADAMTS18*:c.973-1G>C p.?  *AMFR*:c.641delinsTTT p.(His214Leufs*14) *GALT*:c.563A>G p.(Gln188Arg) *ROBO3*:c.571dup p.(Arg191Profs*61) | del(6)(q26q26) |
| 10 | 8 | de novo/ myeloid | e14a2 (67) |  | 46,​XY,​t(9;22)(q34;q11)[20].nuc ish 9q34(ABLx3),​22q11(BCRx2)(BCR con ABLx1)[86/100] | *NPM1*: c.860_863dup p.(Trp288Cysfs*12) *WT1*:c.1141_1144dup p.(Ala382Valfs*4) | *CFTR:*c.2052dup p.(Gln685Thrfs*4)  *HEATR3*:c.1042-2A>G p.?  *HFE*:c.187C>G p.(His63Asp) *NPM1*: c.860_863dup p.(Trp288Cysfs*12) | / |
| 11 | 17 | de novo/ myeloid | e14a2 (77) |  | 46,​XX,​t(9;22)(q34;q11)[3] | *ASXL1:*c.1900_1922del p.(Glu635Argfs*15) | *ASXL1*:c.1900_ 1922del p.(Glu635Argfs*15)  *ATP2B4*:c.1714C>T p.(Arg572Cys) *MTRR*:c.1678_1681del p.(Glu560Asnfs*42) *PIDD1*:c.2042-2A>G p.? *TP63*:c.977C>T p.(Thr326Ile) | / |
| 12 | 15 | secondary/ myeloid | e14a2 (61) |  | 46,​XY,​add(6)(p24),​t(9;22)(q34;q11)[8]/46,​idem,​dup(1)(q21q43)x2,​-add(6)[7].nuc ish 9q34(ABLx3),22q11(BCRx2)(BCR con ABLx1)[90/100] | *ABL1*:c.763G>A p.(Glu255Lys) *RUNX1*:c.597del p.(Arg201Glufs*10)# | *ABCC8:*c.3919C>T p.(Gln1307*)  *ABL1*:c.763G>A p.(Glu255Lys)  *BCHE:*c.428G>A p.(Gly143Asp) *COG7:*c.1498dup p.(Tyr500Leufs*4) *CPXM2:*c.814C>T p.(Arg272Cys)  *FANCM*:c.5791C>T p.(Arg1931*) *OFD1*:c.710dup p.(Tyr238Valfs*2) *RUNX1*:c.597del p.(Arg201Glufs*10)  *SLC26A4*:c.1246A>C p.(Thr416Pro) *WNT10A*:c.682T>A p.(Phe228Ile) | dup(1)(q24.3) dup(3)(q13.31) del(5)(q14.3q14.3) del(7)(p12.1p12.1)/ *IKZF1* deletion# del(7)(q34q34) |
| 13 | 13 | secondary/ lymphoid | e14a2 (42) |  | 46,XX,dup(8)(q24q24),t(9;22)(q34;q11)[15] | *ABL1*:c.763G>A p.(Glu255Lys) *ABL1*:c.944C>T p.(Thr315Ile) | *ABL1*:c.944C>T p.(Thr315Ile)  *NADSYN1*:c.1717G>A p.Ala573Thr | del(7)(p12.3p12.3) del(9)(p13.2p13.2) |
| 14 | 15 | secondary/ lymphoid | e13a2 (39) |  | 46,XY,t(9;22)(q34;q11)[1]/44,idem,add(1)(p22),dic(4;9)(p11;p11),-7[12]/44,idem,add(1)(p22), -7,der(9)t(1;9)(q21;q34),dic(4;9)(p11;p11[2] | *ABL1*:c.763G>A p.(Glu255Lys) *ABL1*:c.944C>T p.(Thr315Ile) *PAX5*:c.77T>G p.(Val26Gly) | *ABL1*:c.763G>A p.(Glu255Lys) *ABL1*:c.944C>T p.(Thr315Ile)  *HFE*:c.845G>A p.(Cys282Tyr) *HPS4*:c.384+1G>A p.? *IRAK3*:c.887+1G>A p.? *PAX5*:c.77T>G p.(Val26Gly) *RECQL4*:c.1573del p.(Cys525Alafs*33)  *SLC35C1*:c.503_505del p.(Phe168del) *TCIRG1*:c.1742T>C p.(Phe581Ser) | del(4)(p11) -7/*IKZF1* deletion# del(9)(p11.1)/ *CDKN2A/B* deletion# |
| 15 | 9 | secondary/ lymphoid | e13a2 (22) |  | 45,XY,-7,i(9)(q10),t(9;22)(q34;q11)[7]/46,XY[13] | / | *FTCD*:c.990dup p.(Pro331Alafs*2) | / |
| 16 | 17 | secondary/ lymphoid | e13a2 (>100) |  | 45~46,​XY,​?dic(7;9)(?q11;q12),​add(10)(q24),​add(11)(q14),​?der(12)t(12;21)(p12;p11),​-14,​-21,​ +der(22)t(9;22)(q34;q11),mar1[cp12]/45~46,​idem,​t(X;11)(p21;q24)[3].​nuc ish 9q34(ABLx3),​22q11(BCRx3) (BCR con ABLx2)[94/100] | *ATM*:c.1179_1180del p.(Trp393*) | *ALMS1*:.3295_3296del p.(Gln1099Lysfs*19)  *ATM*:c.1179_ 1180del p.(Trp393*)  *CFTR*:c.1210-34TG [12]T[5] p.? *CNGB3*:c.819_826del p.(Arg274Valfs*13) *HFE*:c.187C>G p.(His63Asp) | / |
| 17 | 17 | secondary/ lymphoid | e13a2 (81) |  | 45,​XX,​add(7)(p12),​t(9;22)(q34;q11),​add(9)(p12), ​-20[10]/46,​XX[5] | *ABL1*:c.835G>A p.(Glu279Lys) | *ABCC8:*c.1355T>C p.(Leu452Pro)  *ABL1*:c.835G>A p.(Glu279Lys) *ADAMTS13*:c.3178C>T p.(Arg1060Trp) *EPG5*:c.7328del p.(Val2443Alafs*63) *GALT*:c.563A>G p.(Gln188Arg) *MYH2*:c.737G>A p.(Arg246His)  *RAD54B*:c.247del p.(Ser83Valfs*14) | del(7)(p11.1)/*IKZF1* deletion# del(9)(p13.2)/ *CDKN2A/B* deletion# del(20)(q11.2q13.13) |
| 18 | 15 | secondary/ lymphoid | e13a2/ e14a2 (>100) |  | 45,​XX,​del(9)(p12p13),​t(9;22)(q34;q11),​dic(13;20)(p11;q12)[9]/46,​idem,​+der(22)t(9;22)[5]/ 46,​XX[1].​nuc ish 9q34(ABLx3),​22q11(BCRx2) (BCR con ABLx1)[69/100] | / | *CFTR:*c.1521_1523del p.(Phe508del)  *CHEK2*:c.1100del p.(Thr367Metfs*15)  *MPO*:c.1705C>T p.(Arg569Trp) *PKHD1L1*:c.7997G>T p.(Gly2666Val) *SLC6A19*:c.517G>A p.(Asp173Asn) *SORD*:c.757del p.(Ala253Glnfs*27) *TRAF3IP1*:c.799C>T p.(Arg267*) | del(9)(p13.p21.3)/ *CDKN2A/B* deletion# del(20)(q11.21q13) |
| 19 | 18 | secondary/ lymphoid | e14a2 (63) |  | 46,XY,der(7;9)(q10;q10)t(9;22)(q34;q11), der(22)t(9;22),+der(22)t(9;22)[14]/46,XY[3].nuc ish 9q34(ABL1x3),22q11(BCRx4)(BCR con ABL1x2)[67/100] | *SETD2*:c.4715+1G>T p.? *SETD2*:c.3862C>T p.(Gln1288*) | *BTD:*c.1395C>G p.(His465Gln) *CFI:*c.772G>A p.(Ala258Thr) *HEXA:*c.1361G>C p.(Gly454Ala) *HFE:*c.187C>G p.(His63Asp)  *SETD2*:c.4715+1G>T p.? *SETD2*:c.3862C>T p.(Gln1288*) | del(7)(p11.2)/*IKZF1* deletion# del(9)(p11.1)/ *CDKN2A/B* deletion# dup(9)(q34.11) dup(22)(q11) |
|  |  |  |  |  |  |  |  |  |

# RAG-mediated events: *IKZF1* deletion*,* pathogenic *RUNX1* variants*, CDKN2A/B*  deletion

**Table S2: Detailed information for all presented variants.** All variants were classified according to the ACMG/AMP criteria and, if applicable, ClinGen Variant Curation Expert Panel specifications were applied.

| **UPN** | **Gene (transcript)** | **Variant** | **VAF%** | **Variant origin** |
| --- | --- | --- | --- | --- |
| 1 | *ABL1*  (NM_005157.6) | c.1375G>A  p.(Glu459Lys) | 26 | somatic |
|  | *ALOX12B*  (NM_001139.3) | c.340C>T p.(Arg114Trp) | 36 | germline |
|  | *MANBA*  (NM_005908.4) | c.731C>G p.(Ser244*) | 59 | germline |
|  | *NBN*  (NM_002485.5) | c.657_661del  p.(Lys219Asnfs*16) | 60 | germline |
|  | *PRORP*  (NM_014672.4) | c.1064_1065del p.(Ile355Argfs*11) | 43 | germline |
| 2 | *ASXL1*  (NM_015338.6) | c.1773C>A p.(Tyr591*) | 29 | somatic |
|  | *CFTR*  (NM_000492.4) | c.1210-34TG [12]T[5] p.? | 48 | germline |
|  | *COCH*  (NM_004086.3) | c.263G>T p.(Gly88Val) | 63 | germline |
|  | *GJB2*  (NM_004004.6) | c.35del p.(Gly12Valfs*2) | 59 | germline |
|  | *HERC2*  (NM_004667.6) | c.1285G>A p.(Gly429Arg) | 51 | germline |
|  | *HFE*  (NM_000410.4) | c.187C>G p.(His63Asp) | 53 | germline |
|  | *MEI1*  (NM_152513.4) | c.938del p.(Pro313Glnfs*36) | 47 | germline |
| 3 | *CCDC34*  (NM_030771.2) | c.498+1G>A p.? | 40 | germline* |
|  | *CFTR*  (NM_000492.4) | c.1210-34TG [12]T[5] p.? | 46 | germline* |
|  | *CYP27A1*  (NM_000784.4) | c.1342C>T p.(Arg448Cys) | 57 | germline* |
|  | *DNAH8*  (NM_001206927.2) | c.12496C>T p.(Arg4166*) | 46 | germline* |
|  | *LAMA2*  (NM_000426.4) | c.6196A>T p.(Lys2066*) | 39 | germline* |
|  | *RUNX1*  (NM_001754.5) | c.319C>T p.(Arg107Cys) | 42 | somatic* |
| 4 | *CFTR*  (NM_000492.4) | c.1210-34TG[12]T[5] p.? | 46 | germline |
|  | *FAM161A*  (NM_001201543.2) | c.1751+1G>A  p.? | 58 | germline |
|  | *SYNE4*  (NM_001039876.3) | c.129-1G>T p.? | 59 | germline |
| 5 | *CHST14*  (NM_130468.4) | c.653G>T p.(Arg218Leu) | 42 | germline* |
|  | *FLG*  (NM_002016.2) | c.1501C>T p.(Arg501*) | 64 | germline* |
|  | *PCLO*  (NM_033026.6) | c.7325dup p.(Leu2443Valfs*15) | 16 | somatic* |
|  | *STAC3*  (NM_145064.3) | c.862A>T p.(Lys288*) | 63 | germline* |
| 6 | *NDUFS1* | c.968T>G p.(Leu323Arg) | 47 | germline |
|  | *SLC19A1*  (NM_194255.4) | c.274T>C p.(Tyr92His) | 34 | germline |
| 7 | *CBS*  (NM_000071.3) | c.833T>C p.(Ile278Thr) | 30 | somatic |
|  | *GFPT1*  (NM_001244710.2) | c.686-2A>G p.? | 53 | germline |
|  | *GYS2*  (NM_021957.4 | c.547C>T p.(Gln183*) | 39 | germline |
|  | *HFE*  (NM_000410.4) | c.187C>G p.(His63Asp) | 61 | germline |
|  | *NOX3*  (NM_015718.3) | c.1159G>A p.(Gly387Arg) | 53 | germline |
|  | *NR2E3*  (NM_014249.4) | c.227G>A p.(Arg76Gln) | 52 | germline |
| 8 | *BCKDHB*  (NM_183050.4) | c.1149T>A p.(Tyr383*) | 64 | germline |
|  | *CFTR*  (NM_000492.4) | c.1210-34TG [12]T[5] p.? | 55 | germline |
|  | *CLCN1*  (NM_000083.3) | c.950G>A p.(Arg317Gln) | 44 | germline |
|  | *CRELD1*  (NM_001077415.3) | c.927T>A p.(Cys309*) | 49 | germline |
|  | *NPHP3*  (NM_153240.5) | c.3373C>T p.(Arg1125*) | 54 | germline |
| 9 | *ADAMTS18*  (NM_199355.4) | c.973-1G>C p.? | 63 | germline |
|  | *AMFR*  (NM_001144.6) | c.641delinsTTT p.(His214Leufs*14) | 57 | germline |
|  | *GALT*  (NM_000155.4) | c.563A>G p.(Gln188Arg) | 50 | germline |
|  | *ROBO3*  (NM_022370.4) | c.571dup p.(Arg191Profs*61) | 61 | germline |
| 10 | *CFTR*  (NM_000492.4) | c.2052dup p.(Gln685Thrfs*4) | 48 | germline |
|  | *HEATR3*  (NM_182922.4) | c.1042-2A>G  p.? | 94 | somatic |
|  | *HFE*  (NM_000410.4) | c.187C>G p.(His63Asp) | 40 | germline |
|  | *NPM1*  (NM_002520.7) | c.860_863dup  p.(Trp288Cysfs*12) | 18 | somatic |
|  | *WT1*  (NM_024426.6) | c.1141_1144dup p.(Ala382Valfs*4) | 5 | somatic |
| 11 | *ASXL1*  (NM_015338.6) | c.1900_1922del p.(Glu635Argfs*15) | 31 | somatic*^2^ |
|  | *ATP2B4*  (NM_001684.5) | c.1714C>T p.(Arg572Cys) | 45 | germline |
|  | *MTRR*  (NM_002454.3) | c.1678_1681del p.(Glu560Asnfs*42) | 58 | germline |
|  | *PIDD1*  (NM_145886.4) | c.2042-2A>G  p.? | 19 | somatic |
|  | *TP63*  (NM_003722.5) | c.977C>T p.(Thr326Ile) | 59 | germline |
| 12 | *ABCC8*  (NM_000352.6) | c.3919C>T p.(Gln1307*) | 37 | germline |
|  | *ABL1*  (NM_005157.6) | c.763G>A p.(Glu255Lys) | 48 | somatic |
|  | *BCHE*  (NM_000055.4) | c.428G>A p.(Gly143Asp) | 44 | germline |
|  | *COG7*  (NM_153603.4) | c.1498dup p.(Tyr500Leufs*4) | 44 | germline |
|  | *CPXM2*  (NM_198148.3) | c.814C>T p.(Arg272Cys) | 43 | germline |
|  | *FANCM*  (NM_020937.4) | c.5791C>T p.(Arg1931*) | 53 | germline |
|  | *OFD1*  (NM_003611.3) | c.710dup p.(Tyr238Valfs*2) | 15 | somatic |
|  | *RUNX1*  (NM_001754.5) | c.597del p.(Arg201Glufs*10) | 44 | somatic |
|  | *SLC26A4*  (NM_000441.2) | c.1246A>C p.(Thr416Pro) | 41 | germline |
|  | *WNT10A*  (NM_025216.3) | c.682T>A p.(Phe228Ile) | 55 | germline |
| 13 | *ABL1*  (NM_005157.6) | c.763G>A p.(Glu255Lys) | 1 | somatic |
|  | *ABL1*  (NM_005157.6) | c.944C>T p.(Thr315Ile) | 13 | somatic |
|  | *NADSYN1*  (NM_018161.5) | c.1717G>A p.Ala573Thr | 46 | germline |
| 14 | *ABL1*  (NM_005157.6) | c.763G>A p.(Glu255Lys) | 21 | somatic |
|  | *ABL1*  (NM_005157.6) | c.944C>T p.(Thr315Ile) | 11 | somatic |
|  | *HFE*  (NM_000410.4) | c.845G>A p.(Cys282Tyr) | 48 | germline |
|  | *HPS4*  (NM_022081.6) | *c.384+1G>A p.?* | 41 | germline |
|  | *IRAK3*  (NM_007199.3) | c.887+1G>A p.? | 46 | germline |
|  | *PAX5*  (NM_016734.3) | c.77T>G  p.(Val26Gly) | 13 | somatic |
|  | *RECQL4*  (NM_004260.4) | c.1573del p.(Cys525Alafs*33) | 50 | germline |
|  | *SLC35C1*  (NM_018389.5) | c.503_505del p.(Phe168del) | 50 | germline |
|  | *TCIRG1*  (NM_006019.4) | c.1742T>C p.(Phe581Ser) | 52 | germline |
| 15 | *FTCD*  (NM_206965.2) | c.990dup p.(Pro331Alafs*2) | 39 | germline |
| 16 | *ALMS1*  (NM_001378454.1) | c.3295_3296del p.(Gln1099Lysfs*19) | 46 | germline |
|  | *ATM*  (NM_000051.4) | c.1179_1180del p.(Trp393*) | 46 | germline |
|  | *CFTR*  (NM_000492.4) | c.1210-34TG [12]T[5] p.? | 46 | germline |
|  | *CNGB3*  (NM_019098.5) | c.819_826del p.(Arg274Valfs*13) | 67 | germline |
|  | *HFE*  (NM_000410.4) | c.187C>G p.(His63Asp) | 44 | germline |
| 17 | *ABCC8*  (NM_000352.6) | c.1355T>C p.(Leu452Pro) | 68 | germline |
|  | *ABL1*  (NM_005157.6) | c.835G>A p.(Glu279Lys) | 9 | somatic |
|  | *ADAMTS13*  (NM_139027.6) | c.3178C>T p.(Arg1060Trp) | 64 | germline |
|  | *EPG5*  (NM_020964.3) | c.7328del p.(Val2443Alafs*63) | 59 | germline |
|  | *GALT*  (NM_000155.4) | c.563A>G p.(Gln188Arg) | 48 | germline |
|  | *MYH2*  (NM_017534.6) | c.737G>A p.(Arg246His) | 56 | germline |
|  | *RAD54B* (NM_012415.3) | c.247del p.(Ser83Valfs*14) | 62 | germline |
| 18 | *CFTR*  (NM_000492.4) | c.1521_1523del p.(Phe508del) | 74 | germline |
|  | *CHEK2*  (NM_007194.4) | c.1100del p.(Thr367Metfs*15) | 54 | germline |
|  | *MPO*  (NM_000250.2) | c.1705C>T p.(Arg569Trp) | 47 | germline |
|  | *PKHD1L1*  (NM_177531.6) | c.7997G>T p.(Gly2666Val) | 49 | germline |
|  | *SLC6A19*  (NM_001003841.3) | c.517G>A p.(Asp173Asn) | 36 | germline |
|  | *SORD*  (NM_003104.6) | c.757del p.(Ala253Glnfs*27) | 55 | germline |
|  | *TRAF3IP1*  (NM_015650.4) | c.799C>T p.(Arg267*) | 52 | germline |
| 19 | *BTD*  (NM_001370658.1) | c.1395C>G p.(His465Gln) | 44 | germline |
|  | *CFI* | c.772G>A p.(Ala258Thr) | 61 | germline |
|  | *HEXA*  (NM_000520.6) | c.1361G>C p.(Gly454Ala) | 54 | germline |
|  | *HFE*  (NM_000410.4) | c.187C>G p.(His63Asp) | 52 | germline |
|  | *SETD2*  (NM_014159.7) | c.4715+1G>T  p.? | 29 | somatic |
|  | *SETD2*  (NM_014159.7) | c.3862C>T  p.(Gln1288*) | 47 | somatic |

* no material for germline testing, but VAF is low, *^2^ VAF reduced in molecular response material compared to VAF in blast phase material

**Table S3: Characteristics of a comparison cohort of (n=19) pediatric patients with CML-CP**

| **UPN** | **Gender** | **Age at diagnosis of chronic phase** | ***BCR::ABL1* fusion transcript** | ***BCR::ABL1* transcript ratio at diagnosis (%)** | **Karyotype at diagnosis (chronic phase)** | **Targeted subpanel with genes involved in DNA damage response (DDR) (list was generated based on an NCBI search with the terms: “DNA repair”, “DNA damage response”, “DNA replication”, “telomere-associated genes”)** |
| --- | --- | --- | --- | --- | --- | --- |
| 1a | m | 14 | e13a2 | 27,92 | 46,​XY,​t(9;22)(q34;q11)[15]. nuc ish 9q34(ABLx3),​22q11(BCRx2) (BCR con ABLx1)[100/100] | no pathogenic variants detected |
| 2a | f | 7 | e13a2/e14a2 | >100 | 46,​XX,​t(9;22)(q34;q11)[15]. nuc ish 9q34(ABLx3),​22q11(BCRx2)(BCR con ABLx1)[95/100] | no pathogenic variants detected |
| 3a | m | 15 | e13a2/e14a2 | >100 | 46,​XY,​t(9;22)(q34;q11),inc[15] | no pathogenic variants detected |
| 4a | m | 13 | e14a2 | 23,80 | 46,​XY,​t(9;22)(q34;q11)[13]/46,​XY[2]. nuc ish 9q34(ABLx2~3),​​22q11(BCRx2) (BCR con ABLx1)[93/100] | no pathogenic variants detected |
| 5a | f | 10 | e14a2 | 93,50 | 46,​XX,​t(9;22)(q34;q11)[15].​ nuc ish 9q34(ABLx3),​22q11(BCRx2)(BCR con ABLx1)[98/100] | no pathogenic variants detected |
| 6a | m | 16 | e14a2 | >100 | 46,​​XY,​​t(9;22)(q34;q11)[15]. ​nuc ish 9q34(ABLx3),​22q11(BCRx2)(BCR con ABLx1)[93/100] | no pathogenic variants detected |
| 7a | f | 14 | e14a2 | >100 | 46,​XX,​t(9;22)(q34;q11)[15] | no pathogenic variants detected |
| 8a | f | 15 | e14a2 | 46,00 | ​ 46,​XX,​t(9;22)(q34q11)[15].​ nuc ish 9q34(ABLx3),​22q11(BCRx2)(BCR con ABLx1)[96/100] | no pathogenic variants detected |
| 9a | m | 16 | e14a2 | 74,20 | 46,​XY,​t(9;22)(q34;q11)[15].​ nuc ish 9q34(ABLx3),​​22q11(BCRx2)(BCR con ABLx1)[96/100] | no pathogenic variants detected |
| 10a | m | 14 | e14a2 | 45,90 | 46,​XY,​t(9;22)(q34;q11)[17]/46,​XY[3].​ nuc ish 9q34(ABLx3),​22q11(BCRx2)(BCR con ABLx1)[95/100] | no pathogenic variants detected |
| 11a | m | 15 | e14a2 | >100 | 46,​XY,​t(9;22)(q34;q11)[20].​ nuc ish 9q34(ABLx3),​22q11(BCRx2)(BCR con ABLx1)[86/100] | no pathogenic variants detected |
| 12a | f | 16 | e13a2/e14a2 | >100 | 46,​​XX,​​t(9;22)(q34;q11)[20].​ nuc ish 9q34(ABLx2),​22q11(BCRx2)(BCR con ABLx1)[99/100] | *FANC*:c.844-1G>C |
| 13a | m | 6 | e13a2 | >100 | 46,​​XY,​​t(4;9;22)(p15;q34;q11)[15].​ nuc ish 9q34(ABLx3),​​22q11(BCRx2)(BCR con ABLx1)[94/100] | no pathogenic variants detected |
| 14a | m | 15 | e13a2/e14a2 | >100 | 46,​XY,​t(9;22)(q34;q11)[15].​ nuc ish 9q34(ABLx3),​​22q11(BCRx2)(BCR con ABLx1)[94/100] | no pathogenic variants detected |
| 15a | m | 16 | e14a2 | 99,68 | 46,​XY,​inv(9)(p12q21)c,​t(9;22)(q34q11)[15].​ nuc ish 9q34(ABLx3),​22q11(BCRx2)(BCR con ABLx1)[95/100] | no pathogenic variants detected |
| 16a | m | 15 | e14a2 | 54,53 | ​ 46,​XY,​t(9;22)(q34;q11)[15].​ nuc ish 9q34(ABLx3),​​22q11(BCRx2) (BCR con ABLx1)[100/100] | no pathogenic variants detected |
| 17a | m | 8 | not known | >100 | 46,​XY,​t(9;22)(q34;q11)[15].​ nuc ish 9q34(ABLx3),​22q11(BCRx2)(BCR con ABLx1)[95/100] | no pathogenic variants detected |
| 18a | f | 10 | e13a2 | >100 | 46,​XX,​t(9;22;17)(q34;q11;p13)[15].​ nuc ish 9q34(ABLx3),​22q11(BCRx2) (BCR con ABLx1)[100/100] | no pathogenic variants detected |
| 19a | m | 16 | e14a2 | 89,07 | 46,​​XY,​​t(9;22)(q34;q11)[15].​nuc ish 9q34(ABLx3),​22q11(BCRx2)(BCR con ABLx1)[98/100] | no pathogenic variants detected |
